# Primary Mesangial Proliferative Glomerulonephritis: The Forgotten Glomerulopathy

**DOI:** 10.3390/antib15050096

**Published:** 2026-10-12

**Authors:** Edward J. Filippone, John L. Farber

**Affiliations:** 1 Division of Nephrology, Department of Medicine, Sidney Kimmel Medical College, Thomas Jefferson University, Philadelphia, PA 19107, USA; 2 Department of Pathology, Sidney Kimmel Medical College, Thomas Jefferson University, Philadelphia, PA 19107, USA; john.farber@jefferson.edu

**Keywords:** mesangial proliferative glomerulonephritis, IgM nephropathy, C1q nephropathy, rituximab, obinutuzumab, podocytopathy, minimal change disease, focal segmental glomerulosclerosis

## Abstract

Primary mesangial proliferative glomerulonephritis (MesPGN) refers to the light microscopic description of increased mesangial cells (greater than three cells per mesangial area) without endocapillary proliferation, necrosis, and crescents in the absence of another primary glomerulopathy (e.g., IgA nephropathy) or systemic illness (e.g., lupus). Immunofluorescence may be negative or contain mesangial IgM +/− complement (IgM nephropathy) or C1q + immunoglobulin (C1q nephropathy). The presence of either immune deposits or mesangial proliferation per se connotes a worse prognosis when compared with minimal changes. Segmental sclerosis may be present and imparts a worse prognosis regardless of mesangial proliferation or immune deposits. Primary MesPGN may present clinically as nephrotic syndrome, non-nephrotic proteinuria +/− hematuria, or isolated hematuria. If nephrotic, MesPGN may be considered nosologically as a primary podocytopathy with secondary mesangial cell activation, especially when immunofluorescence is negative. Such podocyte injury may be driven by circulating factors and/or antibodies directed against podocyte-specific antigens. Alternatively, IgM or complement deposits suggest an antibody-driven immune-complex etiology primarily affecting the mesangium with secondary podocyte injury analogous to IgA nephropathy, especially when presenting as non-nephrotic proteinuria or hematuria. No randomized controlled trials have addressed immunosuppression in primary MesPGN. If non-nephrotic or isolated hematuria, supportive care is indicated. If nephrotic, initial immunosuppression should mimic that of the primary podocytopathies (high-dose oral steroids), although tacrolimus may substitute if there is a concern for steroid toxicity. For frequently relapsing/steroid-dependent cases, rituximab is our choice for adults, probably with maintenance dosing. Steroid-resistant cases may be treated with calcineurin inhibition.

## 1. Introduction

Mesangial proliferative glomerulonephritis (MesPGN) refers to the light microscopic (LM) finding of mesangial hypercellularity, currently defined as greater than three mesangial cell nuclei per mesangial area [1], with or without increased mesangial matrix. Endocapillary hypercellularity, glomerular necrosis, or crescents should not be present. Mesangial proliferation can be focal or diffuse, segmental or global. There has been inconsistency in the minimum number of mesangial cells per mesangial area required for the diagnosis of MesPGN; any discrepancies versus the current definition will be highlighted when the individual studies are discussed. After utilizing immunofluorescence microscopy (IFM) and electron microscopy (EM), mesangial proliferation may be found in various primary renal diseases, most notably IgA nephropathy (IgAN) [2], or result from systemic illness, such as lupus nephritis, IgA vasculitis with nephritis [3], monoclonal gammopathy of renal significance [4], and post-infectious glomerulonephritis [5]. Other primary glomerulopathies showing mesangial proliferation on LM include fibrillary glomerulonephritis [6] and C3-glomerulopathy [7], but these are readily distinguished by IFM and EM. The topic of this review is primary MesPGN, defined as mesangial proliferation not consistent with IgAN or another primary renal disease and which is unassociated with infection or systemic illness.

A current biopsy report may read MesPGN. In the absence of the exclusions noted above, the treating nephrologist must consider possible pathophysiology and approach to treatment. Notably, in the KDIGO 2021 guideline on treating glomerular disease [8], a separate section on MesPGN is not included. Knowledge is steadily increasing regarding interactions between mesangial cells and podocytes. In a biopsy showing MesPGN, the primary site of disease may involve either cell type with the other secondarily involved. We attempt to provide insight that can be applied to the individual patient to aid in understanding pathophysiology and specific treatment.

MesPGN was initially highlighted as a separate entity in nephrotic children by White et al. in 1970 [9], although it can occur at any age. Clinically, patients with MesPGN may present as steroid-sensitive (SS) nephrotic syndrome (NS), although relapses are common. However, a significant minority are steroid-resistant (SR) from the outset and may also be unresponsive to additional immunosuppressive agents. Microscopic hematuria is found in most and may occasionally be gross. Other patients present with subnephrotic proteinuria and/or hematuria. Hypertension and reduced GFR are more common with mesangial proliferation than with MCD, as is progression to ESKD.

The relationship of MesPGN to the primary podocytopathies, i.e., MCD and primary focal segmental glomerulosclerosis (FSGS), remains uncertain. In the setting of idiopathic NS (INS), i.e., NS with no secondary cause, some biopsies with mesangial proliferation only show diffuse foot process effacement (FPE) on EM without electron-dense deposits (EDDs) and have no immune deposits by IFM; however, IgM immune deposits by IFM with or without complement are common. Segmental sclerosis may occur regardless of immune deposits or EDDs. Border hypothesized nearly 40 years ago that MesPGN is distinct from the primary podocytopathies [10]. According to his hypothesis, MesPGN may initially have only minimal changes (FPE on EM) or mild mesangial abnormalities on LM. As the disease progresses, more marked mesangial proliferation develops and eventuates in segmental sclerosis. Immune deposits are integrally involved, especially IgM and less commonly IgG, along with complement. Podocytes would be secondarily injured, as opposed to the primary podocytopathies wherein the podocyte is the primary target. Others consider MesPGN to be part of the primary podocytopathies, perhaps a transition state from MCD to FSGS, with supporting evidence coming from experimental animals [11] and human studies [12].

Herein we will define the prognostic and therapeutic implications of finding mesangial proliferation in the biopsies of patients with INS. We will consider both the significance of segmental sclerosis in primary MesPGN, as well as the significance of mesangial proliferation in primary FSGS. The significance of immune deposits, i.e., IgM and complement, will be discussed in detail. Our recommendations regarding therapy will be presented.

## 2. Mesangial Proliferation

Mesangial proliferation has been addressed in the pediatric literature for decades [9,13]. The International Study of Kidney Disease in Children (ISKDC) distinguished the adverse outcomes of segmental sclerosis on LM compared with those with only minimal changes [9,13]. The significance of abnormal mesangial proliferation was noted, and such cases were labeled MesPGN.

The ISKDC evaluated 521 children with INS and, after excluding FSGS, found by LM that 219 had completely normal glomeruli, 98 had focal global sclerosis, 29 had focal tubular changes, 16 had mild mesangial thickening, 27 had what they defined as mild mesangial hypercellularity (three cells per peripheral mesangial area), and 12 had what they defined as diffuse mesangial hypercellularity (≥4 cells per peripheral mesangial area, equivalent to the current definition of abnormal mesangial proliferation) [14]. The latter group had a significantly higher percentage of initial steroid non-responders (45.5%) than the others (4.7–14.8%), although by 1 year there were no significant differences.

Similarly, the Southwest Pediatric Nephrology Study Group evaluated 29 children with INS and underlying diffuse mesangial hypercellularity without segmental sclerosis [15]. Microscopic hematuria was found in 89%, and seven had reduced creatinine clearance. Three were IgM-positive and four contained EDDs. All patients had diffuse FPE (70–80%). Of four re-biopsied, one had FSGS. Overall, 12 of 21 steroid-treated patients had complete remissions and three had partial remissions. All four with 3+ mesangial hypercellularity (≥5 nuclei per mesangial area) were unresponsive, although two subsequently had spontaneous remissions. None of the six steroid-resistant patients responded to alkylating agents.

Alexopoulos et al. studied 28 adult Greek patients with INS and MesPGN, excluding segmental sclerosis, 25 of which received steroids [16]. Initially, about one-third had complete remission, one-third partial remission, and one-third were unresponsive. At a mean of 64 months, 40% were in complete remission, 36% in partial remission, and 24% remained unresponsive. Although there was no control group, these results are inferior to the reported results for treating MCD, again supporting the idea that mesangial proliferation is an adverse finding versus MCD. IgM deposits were present in all nonresponders, although they were also present in about 20% with complete remission. Subsequent biopsies in two nonresponders showed FSGS. Altogether, these studies highlight the adverse effect of mesangial proliferation in both children and adults with INS in the absence of segmental sclerosis.

In a patient with INS and MesPGN on initial biopsy, is the presence of segmental sclerosis relevant? Waldherr et al. evaluated 38 nephrotic children with diffuse mesangial proliferation on biopsy divided into 18 with no segmental sclerosis (group 1) and 20 with associated segmental sclerosis (group 2) [17]. Clinical presentation was similar, although 2/16 group 1 patients responded to steroids and two spontaneously remitted compared with none of the group 2 patients. At final evaluation, 5/18 in group 1 and 10/20 in group 2 progressed to ESKD or had impaired function. Of 11 repeat biopsies in group 1, eight developed segmental sclerosis (FSGS). Interestingly, three of these eight no longer had mesangial proliferation. Overall, most patients (>95%) had hematuria and 70% had segmental sclerosis initially or on repeat biopsy. Hence, segmental sclerosis in the setting of MesPGN portends a worse prognosis.

Does mesangial proliferation portend a worse prognosis if found on an initial biopsy showing FSGS? The evidence is conflicting. Schoeneman et al. assessed the significance of “marked mesangial accentuation or proliferation” in 24 children with steroid-unresponsive NS diagnosed as FSGS [18]. Ten of the 13 with mesangial involvement had reduced glomerular filtration rates (≤90 mL/min/1.73 m^2^) at the end of follow-up (four reaching ESKD) compared with none of 11 without mesangial involvement. Neither group was steroid-responsive. Studying 80 nephrotic adults with FSGS, Ponticelli et al. found that only mesangial proliferation and the lack of remission (complete or partial) with immunosuppression significantly predicted the risk of dying or doubling of serum creatinine [19].

By contrast, Nickavar and Rahbar evaluated 46 pediatric nephrotic FSGS patients and found mesangial hypercellularity did not correlate with steroid response, although there were significant correlations with anemia and hypertension [20]. Schwartz et al. studied 81 adult FSGS patients and found that 16% had mesangial hypercellularity that did not significantly correlate with ESKD [21].

Hence, it remains uncertain whether the presence or absence of mesangial hypercellularity in the setting of INS with segmental sclerosis (FSGS) is as relevant as it is in biopsies with MCD, where it portends a worse prognosis. In our opinion, in a patient with INS, segmental sclerosis is the dominant prognostic pathologic finding, whether present on initial biopsy or only on subsequent biopsy, regardless of the original biopsy being MCD or MesPGN.

## 3. IgM Nephropathy

In patients presenting with INS, LM may show MCD, MesPGN, or FSGS, all with diffuse FPE on EM, although MesPGN may have lesser degrees of FPE. Granular mesangial IgM deposits may be found complicating any of the three LM appearances. Rarely, crescents may be observed. These deposits may be focal or diffuse, global or segmental; however, IgM restricted only to segmental scars does not apply. Complement components (C3 and/or C1q) may be present, although C1q staining ≥2+ defines C1q nephropathy (C1qN, vide infra) regardless of IgM. Other immunoglobulin classes may be present, but IgM must be dominant, especially if IgA is present. Whereas IgM-positive biopsies often have associated mesangial proliferation, well-defined deposits may exist with only minimal changes [22]. Mesangial EDDs may or may not be detectable by EM, but significant subendothelial or subepithelial capillary wall deposits should not be found.

Some argue that biopsies containing IgM represent a separate entity, IgM nephropathy (IgMN), versus classification based purely on LM, i.e., MCD, MesPGN, or FSGS. Unfortunately, there is no consensus definition of IgMN. Some authors accept 1+ IgM staining as a minimum threshold, while others require ≥2+. Some require mesangial EDDs on EM. Some authors accept segmental sclerosis, though cases with IgM restricted to segmental scars are not included. It remains uncertain whether IgMN deserves a separate designation as opposed to the standard classification based on LM alone.

IgM deposits (defined qualitatively) with focal or diffuse mesangial proliferation were first described in 1974 in patients presenting with recurrent macroscopic hematuria or persisting microscopic hematuria +/− low-level proteinuria [23]. C3 and C1q were present in all patients; however, IgA was also present in two-thirds. None developed impaired kidney function. In 1978, two groups simultaneously reported MesPGN with IgM deposits underlying INS or asymptomatic non-nephrotic proteinuria [24,25]; all patients had ≥1+ IgM staining. These seminal papers highlight the variable clinical presentation of patients with mesangial IgM depositions, ranging from hematuria with or without non-nephrotic proteinuria to INS with or without hematuria.

## 4. IgM Deposits: Pathogenicity and Prognostic Relevance

Natural IgM antibodies are polyreactive and germline-derived, produced without prior antigen exposure. In mice, natural IgM antibodies are produced by B-1 cells (including the CD5^+^ B-1a and CD5^−^ B-1b subpopulations) located in tissues lining the peritoneal and pleural cavities, as well as spleen, bone marrow, and lymph nodes [26,27,28]. In humans the source is less certain, but they appear to be produced by B-1-like cells (circulating in blood and possibly in other tissues) and B-2-like cells found in blood, lymph nodes, and spleen [28]. Besides reacting with pathogens as the first line of defense, natural IgM is important for the clearance of apoptotic cells. However, these antibodies may react with self-epitopes expressed on injured tissue, including the kidney, and may activate complement to further injury. Relevant epitopes reactive with these natural IgM antibodies may include cardiolipin and other phospholipids [29].

Strassheim et al. evaluated the role of natural IgM to further glomerular injury in a murine model of FSGS, wherein glomerular IgM, C3, and C4d are readily detectable [30]. Treatment with an anti-CD20 antibody reduced glomerular IgM deposition and significantly attenuated clinical disease. Additionally, B-cell-deficient mice had absent glomerular IgM deposition and significantly less albuminuria, FPE, and glomerular C3 deposition than control mice.

Panzer et al. evaluated the effect of natural IgM to cause and/or contribute to glomerular disease in a non-immune-complex, non-sclerotic mouse model of glomerulopathy and thrombotic microangiopathy resulting from Factor-H deficiency [31]. IgM was deposited in the mesangium and subendothelially, and mice lacking both Factor H and IgM had milder disease. Naturally occurring monoclonal IgM from some clones increased urine albumin in Factor-H/IgM-deficient mice but not in wild-type controls.

Trachtman et al. found deposits of IgM and activated complement fragments (C4d, iC3b/C3d, and human C9 neoepitope) in some glomeruli of 10 patients with FSGS (six primary FSGS, four secondary) [29]. Patients with primary podocytopathies (MCD or FSGS) had elevated plasma levels of C4a and soluble C5b-9. Elevated IgM levels reactive with glomerular endothelial cells were detected by both cell-based assay and ELISA targeting cardiolipin in nephrotic patients. Only manipulated glomerular endothelial cells bound a natural IgM monoclonal antibody as well as IgM from normal serum. The fact that these antibodies only reacted with injured cells suggests that they are not the initiators of injury but may be exacerbators binding to neoepitopes exposed by prior injury.

Altogether, the data indicate a potential pathogenic role for IgM, especially naturally occurring IgM, as a contributor to the pathogenesis of INS, regardless of LM. Though IgM may not initiate injury, it may nevertheless amplify it. However, evaluating the prognostic significance of mesangial IgM deposition in the setting of INS has produced conflicting results. There is marked heterogeneity in populations studied (pediatric versus adult versus mixed), clinical presentations (INS, non-nephrotic proteinuria +/− hematuria), LM findings (MCD, MesPGN, FSGS, or mixtures), EM findings (EDDs required or not, degree of FPE), and definitions of IgM-positivity (≥1+ versus ≥2+ required). Clinical criteria are variable (e.g., INS required or series based solely on pathology). Furthermore, in series based on pathology, the specific biopsy practices of the center may influence the frequency with which less severe clinical manifestations are found, such as non-nephrotic proteinuria and/or hematuria. We will discuss selected studies based on the LM findings and age of presentation (pediatric versus adult), highlighting the inconsistent results that hamper making specific conclusions.

## 5. Childhood IgM Nephropathy

Studies evaluating the significance of IgM deposits in pediatric MCD have been conflicting (see Table 1). Biopsies are typically performed in this population for steroid-dependence (SD) or steroid-resistance (SR) unless patients are <1 year old or >10–12 years old. Pardo et al. evaluated 61 children with MCD (mild mesangial proliferation accepted) and found 36 biopsies from 33 patients had mesangial IgM deposits (24 diffuse/global, 12 focal/segmental) with C3 in 11 [32]. There was no significant difference in therapeutic response based on IgM. Al-Eisa et al. evaluated 27 pediatric patients with diffuse mesangial IgM staining, excluding patients with segmental sclerosis, and compared them with 63 diagnosed as MCD, although mesangial expansion was allowed in either group [33]. There was no difference in baseline characteristics, response to steroids, or outcome between the groups.

Vintar Spreitzer et al. studied 55 children with MCD divided into IFM-negative cases (29/55), IgM-positive cases (19/55), and C1q nephropathy (C1qN, vide infra) defined as C1q ≥ 2+ intensity regardless of other immunoglobulin staining (7/55) [34]. Diffuse mesangial hypercellularity was present in about 10–15% of each group. EDDs were found in 5/13 with IgM deposits and 6/6 with C1qN. There was no difference between these groups in baseline clinical characteristics, clinical course, or kidney outcome after a median follow-up of 16.9 years.

By contrast, others found adverse features associated with IgM deposition in pediatric MCD. Zeis et al. evaluated 85 nephrotic children with MCD (allowing mesangial proliferation) and found 20 had positive IFM, mainly with IgM, and 65 were negative [35]. On follow-up biopsies, 6/20 (30%) IFM-positive patients evolved into FSGS compared with 3/65 (4.6%) IFM-negative patients. Of 331 children biopsied for non-nephrotic proteinuria and/or hematuria, 44 were IgM-positive and 148 were IFM-negative; the remainder had IgA-IgG deposits. Overall, 7/44 (16%) IgM-positive patients evolved into FSGS compared with 0/148 IFM-negative cases.

Other pediatric studies combined patients with MCD, MesPGN, or FSGS by LM and assessed the significance of IgM. Habib et al. assessed 222 French children with INS and found 122 with MCD, 10 with MesPGN, and 90 with FSGS [36]. Of the 222, 54 were IgM-positive, including 33/122 with MCD, 2/10 with MesPGN, 19/90 with FSGS. There was no significant difference in response to steroids based on IgM-positivity. Cases with other immune reactants (C1q, IgG, IgA) also did not respond differently compared with IFM-negative cases.

In a study of 45 Lithuanian children with INS, Juozapaite et al. found 18 were IgM-positive (8 MCD, 1 MesPGN, 9 FSGS based on LM) and 27 IgM-negative (16 MCD, 6 MesPGN, 5 FSGS) [37]. After approximately 4 years of follow-up, there was no significant difference in remission, disease activity, or decrease in kidney function.

Albakr et al. studied 15 Saudi children with IgM-positive biopsies, including 9 MesPGN, 1 FSGS, 4 MCD, and 1 uncertain [38]. Clinically, 14/15 had INS, and all but one responded to steroids initially. Relapses were common. Most were in complete remission at last follow-up with one progressing to ESKD.

By contrast, other studies combining variable LM demonstrated worse outcomes with IgM-positivity. Swartz et al. reviewed biopsies of 170 Texas children and found that 55 with available data were IgM-positive, including (by LM) MCD (23/55 IgM-positive), MesPGN (19/43 IgM-positive), and FSGS (13/72 IgM-positive). Of 23 with IgM-positive MCD, 3 (13%) developed ESKD, a much higher rate than expected for IFM-negative MCD [39]. Adjuvant therapy for IgM-positive MCD was more effective with cyclosporine (7/8 responding to initial therapy, 5/6 as secondary therapy) compared with cyclophosphamide (2/11 responding).

**Table 1:** **Selected series of pediatric IgM nephropathy.**

	Number	Diagnostic Criteria	Light Microscopy	Clinical Manifestations	Treatment	Response	Follow-Up	Comment
Pardo [32] 1984	33 with IgM 28 without	Mesangial IgM ≥ 1+	MCD Or mild MesP	INS: all 33 HU: 7/33 Elevated Cr: 8/33	Prednisone 100% Cyclophos: 8/33	Response: 72% Relapse: 30%	Median 5 years	Mes EDDs in 10 Similar course to 28 IgM- MCD
Habib [36] 1988	54	IgM+ Not defined	MCD: 33 MesPGN: 2 FSGS: 19	INS: all 54	Prednisone	Response: 2 Partial: 8 No response: 19	>1 year up to 20 years	No EDDs in 13 evaluated
Al-Eisa [33] 1996	27 63 with IgM-MCD	Mesangial IgM ≥ 1+	Mesangial expansion	INS: 96% HU: 33% Reduced GFR: 4%	Prednisone: 93%	Response: 91% Relapse: 100%	Mean 32 months	Similar course to 63 IgM- MCD
Zeis [35] 2001	27 IgM+ (INS) 44 IgM+ (PU/HU)	IgM+ Granular, diffuse mesangial IgM	MCD: 20 FSGS: 7	INS PU/HU	Prednisolone	Response: 14/27	1–12 years	30% evolved to FSGS
Swartz [39] 2009	55	IgM+ Not defined	MCD: 23 MesPGN: 19 FSGS: 13	INS: 100% HU: 34/55	SD: 24 SR: 26 Cyclophos: 26 CsA: 21	CsA better response than cyclophos	Mean 64 months; median 30.5 months	18% (10/55) CKD 13% (7/55) ESKD
Mubarak [40] 2011	135	IgM ≥ 2+, diffuse and global	MCD: 46 (34%) MesPGN: 89 (66%) FSGS: 37 (27.4%) *	INS: 100% HU: 42 (31.2%)	Steroids: 100%	Not available	Mean 38.4 months	C3+ in 53% C1q+ in 29.7%
Kanemoto [41] 2013	30		MCD: 21 MesPGN: 6 FSGS: 3	INS: 100% HU: 16.7%	CsA: 22 Mizoribine: 5 Tacrolimus: 1 MMF: 3	CR: 25/30 FR: 2/30 PR: 2/30	Median 5.0 years	Overall, good response to CsA
Spreitzer [34] 2014	19	IgM ≥ 2+	MCD	INS: 100% HU: 71%	Cyclophos: 69% Levamisole: 15% MMF: 15%	SS/IR: 6 FR: 1 SD: 9 SR: 3	Median 16.9 years	No difference versus IgM-MCD
Juozapaite [37] 2017	18	IgM ≥ 1+	MCD: 8 MesPGN: 1 FSGS: 9	INS: 100% HU: 72% Reduced GFR: 11%	Steroids: 100% CsA: 83% MMF: 22% Cyclophos: 33% Rituximab: 17% Levamisole: 17%	Remission: 61% ESKD: 11%	Median 4.52 years	No difference in outcome IgM+ versus IgM- groups
Albakr [38] 2025	15	IgM ≥ 1+	MesPGN: 14 FSGS: 1	INS: 14/15 HU: 2/15	Steroids: 14/15 CNIs: 4/15 MMF: 2/15 Rituximab: 4/15	SS/NR: 1 IR: 1 FR/SD: 6 SR: 5	Median 9.4 years	Overall relapse rate: 93%

CNI: calcineurin inhibitors; CR: complete remission; CsA: cyclosporin A; Cyclophos: cyclophosphamide; EDDs: electron-dense deposits by electron microscopy; FR: frequent relapse; FR/SD: frequently relapsing/steroid-dependent; FSGS: focal segmental glomerulosclerosis by light microscopy; HU: hematuria; INS: idiopathic nephrotic syndrome; IR: infrequent relapse; MCD: minimal change disease; MesPGN: mesangial proliferative glomerulonephritis by light microscopy; MMF: mycophenolate mofetil; NR: no remission; PR: partial remission; PU: proteinuria; SD: steroid-dependent; SR: steroid-resistant; SS: steroid sensitive. * all 37 with FSGS had mesangial proliferation and are part of the 89 with MesPGN.

Mubarak et al. identified 135 pediatric Pakistani patients with INS- and IgM-positive biopsies, including 46 MCD, 52 MesPGN, and 37 FSGS (all 37 with mesangial proliferation) [40]. Comparing a subset of 95 IgM-positive patients to 267 IgM-negative MCD patients showed greater hematuria (28 versus 16%) and progression to kidney failure (16 versus 2.5%), although IgM-positive patients were not restricted to MCD on LM as the controls were.

Kanemoto et al. reviewed 70 Japanese children with steroid-dependent or steroid-resistant INS and found 30 were IgM-positive, including 21 of 53 with MCD, 6 of 10 with MesPGN, and 3 of 7 with FSGS [41]. Overall, the IgM-positive group was more likely steroid-resistant (14/30, 46.7%, versus 11/40, 27.5%, *p* < 0.05). Notably, all 14 steroid-resistant IgM-positive cases responded to cyclosporine, mimicking the results of Swartz noted above.

## 6. Adult IgM Nephropathy

Studies in adults are similar (see Table 2), with results overall suggesting a worse prognosis compared with adult MCD. Saha et al. evaluated 54 patients (46 adults) with diffuse and global mesangial IgM deposits, including 22 with MCD and 34 with MesPGN [42]. Clinically, 18 had INS, 21 non-nephrotic proteinuria, 4 proteinuria/hematuria, and 11 isolated hematuria. Of the 18 with INS, 16 were given steroids, 6 of which were resistant. O’Donoghue et al. followed 54 predominantly adult-aged patients with diffuse, global IgM deposition and MesPGN (segmental sclerosis in 19), including 31 with INS, 19 with asymptomatic proteinuria, and 4 with macroscopic hematuria; 18 of 31 with INS were given high-dose steroids, of which 5 remitted [43]. Eventually, 14 patients developed ESKD between 1 and 10 years; half of the remaining patients were in complete remission, and half had persisting proteinuria. Yang et al. evaluated 37 adults with INS and MCD by LM divided into 12 with IgM deposits and 25 without [44]. The baseline clinical presentation and response to treatment were not different between the two groups, with the exception that the recurrence rate was significantly higher with IgM deposition.

Lee et al. evaluated 63 adult MCD patients and found 15 had positive IFM, including 9 with IgM deposits [45]. Other deposits included C1q in 4, IgG in 4, IgA in 3, and C3 in 1. By multivariable analysis, the presence of deposits was the only significant factor predicting the composite endpoint (doubling of serum creatinine, ESKD, or death), which occurred in 4/15 (26.7%).

Little et al. evaluated 54 predominantly adult patients with MesPGN by LM and diffuse IgM deposits, including 22 with INS, 14 with non-nephrotic proteinuria, with (54%) or without (46%) hematuria, and 18 with isolated hematuria (gross in 10) without proteinuria [46]. Four of the 22 INS patients progressed to ESKD, 2 of whom were re-biopsied and had segmental sclerosis. Of 20 patients with INS given steroids, 12 had remission, 10 of whom became steroid-dependent. Of 11 receiving cyclophosphamide, 3 had lasting response, 6 relapsed upon stopping therapy, and 2 had no response. Of 10 given cyclosporine, 2 had a lasting response, 5 relapsed after remitting, and 3 had no response. None of the patients with non-nephrotic proteinuria or isolated hematuria were immunosuppressed and none had progressive loss of kidney function over 7 years. There was no control group that had MesPGN without IgM.

**Table 2:** **Selected series of IgM nephropathy in adults.**

	Number	Diagnostic Criteria	Light Microscopy	Clinical Manifestations	Treatment	Follow-Up	Response	Comment
Saha [42] 1989	54 *	Diffuse, granular IgM	MCD: 22 Mild MH: 32	INS: 18 * PU: 21 PU/HU: 4 HU: 11	INS: steroids: 16/18	5 years	SS: 2 SD: 8 SR: 6	Nine of 18 with INS had decline in renal function, 3 ESKD
O’Donoghue [43] 1991	54	Diffuse, global MH with IgM predominant	MesPGN: all FSGS: 19	INS: 31 PU: 19 HU: 18 (33%) **	INS: 18/31 high-dose steroids	Minimum of 3 years or ESKD	5: remission 2: partial remission	Included IgA+: 9 8 ESKD by 3 years, 14 ESKD eventually
Lee [45] 2016	9	IgM: ≥ 2+ EDDs or MME	MCD: all	INS: all	NA	Median 5.0 years	NA	Immune deposits significantly associated with doubling creatinine or ESKD versus no deposits
Connor [47] 2017	57	IgM: >trace and predominant EDDs required	MCD: 13 MH/MME: 39 FSGS: 40	INS: 22 PU: 35	INS: Prednisolone: 11 Other: 17 ***	Median 40 months	PR: 14 CR: 9	Ten rebiopsied, only 1 IgMN (EDD+), 1 MCD, 8 FSGS
Little [46] 2000	54	Diffuse MesPGN with diffuse mesangial IgM	MesPGN: all	INS: 22 (41%) PU: 26% HU: 33%	INS: Steroids20/22 CycloP: 11/22 CsA: 10/22	Mean 7.4 years	Remission: Steroids: 12/20 CycloP: 3/11 CsA: 2/10	Four reached ESKD, 2 of which were rebiopsied: FSGS
Yang [44] 2021	12	IgM ≥ 1+	MCD: all	INS: all	Prednisolone: 11 Other: 7 ****	2 years	CR: 10/12 NR: 2/12	Significantly greater relapse rate versus IgM-MCD
Chae [48] 2021	94	IgM ≥ 1+ without any other immunoglobulin	MCD: 25 MesPGN: 48 FSGS: 21	NRP: 36% (not in MesPGN) HU: 62%	Steroids: 39% Other: 6.5%	Mean 22.5 months	NA	IgM+ group as a whole fared similar to IgM-MCD, IgM-MesPGN, IgM-FSGS
Yun [49] 2025	63	IgM > trace and predominant EDDs+ in 13 EDDs- in 50	MH: 62%/26% ***** MME: 54%/20% SS: 3%/7%	NRP: 15%/35% HU: 54%/55%	Steroids: 21%/36% Other: 23%/32%	Median 79.5 months	NA	~50% >50% decline in eGFR or ESKD similar based on EDDs

*: included 8 children. **: 4 macroscopic. ***: tacrolimus in 10, mycophenolate in 3, rituximab in 3, cyclophosphamide in 1. ****: cyclosporine 5, mycophenolate 1, cyclophosphamide 1. *****: light microscopy and clinical findings characterized by EDD+ versus EDD−. CR: complete remission; CsA: cyclosporin A; cycloP: cyclophosphamide; EDDs: electron-dense deposits by electron microscopy; FSGS: focal segmental glomerulosclerosis; HU: hematuria; INS: idiopathic nephrotic syndrome; MCD: minimal change disease; MH: mesangial hypercellularity; MME: mesangial matrix expansion by light or electron microscopy; NA: not available; NR: no remission; PR: partial remission; PU: proteinuria; SD: steroid-dependent; SR: steroid-resistant; SS: segmental sclerosis.

Connor et al. evaluated 57 British adult cases with predominant mesangial IgM staining accompanied by mesangial EDDs in all cases [47]. By LM, 13 (23%) had MCD and 40 had FSGS (70%); 17 (30%) were found with mesangial proliferation alone or with FSGS. Less than half (23/57) had nephrotic syndrome, 26 (45.6%) had non-nephrotic proteinuria, 6 presented with hematuria, and 3 had declining kidney function at the time of biopsy. Of 23 nephrotic patients, 14 achieved partial remission with 9 achieving complete remission. Doubling of serum creatinine occurred in 25% at 3 years and 30% at 5 years. Seven reached ESKD with 6/7 having segmental sclerosis on >20% of glomeruli as the worst histologic prognostic feature. There was no adverse effect of mesangial proliferation. Ten patients were re-biopsied, with 8 showing FSGS.

Chae et al. evaluated 94 adult Korean patients with IgMN, including 25 MCD, 48 MesPGN, and 21 FSGS [48]. All 25 with MCD had INS with a urine protein/creatinine ratio (UPCR) of 6.3, but proteinuria levels were significantly lower with MesPGN (UPCR of 0.4, *p* < 0.001) and trended less with FSGS (UPCR 2.1, *p* = 0.089). However, the LM subtype predicted neither eGFR nor proteinuria evolution over time. When compared with IgM-negative patients with MCD, MesPGN, or FSGS, the IgM-positive group considered as a whole and the 3 IgM-negative subgroups considered separately all fared the same.

Yun et al. evaluated 63 adult patients with IgMN defined as dominant mesangial IgM including cases with segmental sclerosis [49]. Of 63 patients, 13 had EDDs in addition to IgM with no significant difference in outcome compared with the 50 without EDDs. The full 63 were then compared with 469 with IgAN, 103 with IgM-negative FSGS, and 91 with IgM-negative MCD. The IgMN group had a similar percentage of glomeruli with segmental sclerosis (6.4%) as the FSGS patients (8.7%, *p* = 0.13), and a similar percentage reached the primary adverse renal outcome of >50% decrease in eGFR, eGFR < 15, or ESKD (46% for IgMN versus 41% for FSGS, *p* = 0.52). These data indicate that in the presence of segmental sclerosis, the presence of IgM did not impact prognosis. IgMN patients overall (MCD and FSGS grouped together) fared significantly worse than MCD patients without IgM deposits considered alone (46% versus 19% reaching renal endpoint, *p* < 0.001); however, the apparent adverse effect of IgM deposits with MCD compared with MCD alone could not be assessed as the IgMN-positive group included those with segmental sclerosis.

Combining children and adults, the presence of diffuse mesangial IgM-positivity is most likely an adverse prognostic factor in those without segmental sclerosis on initial biopsy. Although some studies were negative, others were positive for a worse outcome. No study showed a better outcome with IgM deposits. The conclusions are further hampered by lack of comparability, as IgM-positive cases with variable LM including FSGS were combined in some studies and not separated by LM when compared with LM-specific IgM-negative controls (e.g., IgM-positive patients including FSGS histology compared with only IgM-negative MCD controls). Comparison of studies is hampered by heterogeneity in inclusion criteria. Biopsy practices at individual centers differ, which may affect the frequency of less severe clinical presentations, such as non-nephrotic proteinuria or microscopic hematuria. Thresholds of IgM-positivity differ (≥1+ versus ≥2+ versus unspecified). Some studies require EDDs on EM, though most do not. Some studies excluded cases with segmental sclerosis, while others included them.

The progression to segmental sclerosis on repeat biopsies in those with MCD or MesPGN initially appears more frequent with IgM-positivity. The presence of such sclerosis on repeat biopsy typically results in a diagnosis of FSGS, implying that the diagnosis has changed. In our opinion, the presence of sclerosis on repeat biopsy confirms the worse prognosis suggested by the clinical course necessitating the repeat biopsy, and it does not indicate transition to a new disease. Such a course may complicate IFM-negative MCD or cases with IgM-positivity. Since this appears to occur more frequently when IgM-positive, it lends further credence to the adverse significance of IgM deposits compared with IFM-negative MCD. Whether IgM-positivity imparts worse prognosis in the presence of segmental sclerosis on initial biopsy is less certain. Meta-analyses of these issues are precluded by the marked heterogeneity in the characteristics of the cited studies.

## 7. IgM in Segmental Scars

The presence of IgM +/− C3 restricted to segmental scars does not define IgMN and has been considered of no pathogenic significance. However, Zhang et al. evaluated 106 adult patients with primary FSGS and found that 58 (54.7%) had IgM deposits mainly restricted to segmental scars, a minority of which were C3-positive [50]. None of the IgM-negative patients were complement-positive. Only patients with dual positivity (IgM and C3) were at higher risk for refractory NS (defined as non-remitting or SD), with an odds ratio of 11.32 (*p* = 0.003); those with IgM alone were not. Regarding renal dysfunction, the presence of IgM significantly increased risk regardless of C3 deposition. Importantly, IgM-positivity was mainly restricted to scars and was not present diffusely in the mesangium, suggesting that IgM and complement may be amplifiers of disease in FSGS even when restricted to segmental scars.

Similarly, Mirioglu et al. evaluated 86 Turkish adult primary FSGS patients for the IgM +/− C3 deposits and found 22 had both (dual positivity), 22 had only IgM and 42 had neither [51]. However, IgM-positivity could include deposits either restricted to scars or involving unaffected mesangium. Patients with dual positivity had higher proteinuria, lower eGFR, and a greater degree of segmental sclerosis compared with the other two groups which did not differ. Dual-positive patients more commonly reached the primary endpoint (≥50% decline in eGFR or ESKD, hazard ratio 3.36, *p* = 0.009); they also had lower complete/partial remissions.

By contrast, Pacic et al. studied adult patients with primary FSGS and found no significant relationship between dual IgM and C3 positivity with a composite of ≥50% decline in eGFR, ESKD, or death, although IgM did impart a worse prognosis in secondary FSGS cases [52]. Peng et al. studied 264 Chinese primary FSGS patients and found neither C3 nor IgM deposition considered separately predicted an adverse renal outcome (≥50% reduction of eGFR, ESKD, death from kidney disease), although C3 deposits in the capillary wall in addition to mesangium did [53]. IgM and C3 together were not considered. Looking at 175 patients with primary FSGS, the CureGn Consortium found neither C3 nor IgM staining predicted the composite outcome (≥40% decline in eGFR or ESKD) by multivariable analysis, but complement activation as assessed by urinary soluble C5b-9 did, mimicking the work of prior authors [54]. Such complement activation further implicates an active pathogenic role for IgM, although both animal [55,56] and human studies [54,57] of FSGS also indicate a role for an alternate pathway of complement activation in addition to the IgM-mediated complement activation which can occur by either the classic or lectin pathways [58]. Hence, all three pathways of complement activation may be involved.

Overall, these data support a pathogenic role for IgM in the primary podocytopathies, including MesPGN, presumably through activation of complement. In IgM-positive MesPGN, one or more undefined mesangial antigens may be the primary target of IgM antibodies initiating the disease with secondary podocyte injury. IgM may also be secondarily involved as an amplifier of disease by reacting to neoepitopes exposed by mesangial injury initiated by other mechanisms. It remains uncertain to what degree any detectable IgM is composed of naturally occurring, low-affinity, polyreactive IgM versus T-cell-dependent, high-affinity IgM. To make this distinction would require eluting the antibody and preferably combining a polyreactivity panel, affinity assessment (e.g., using surface plasmon resonance [59]), and V-gene sequence analysis (to assess somatic hypermutation) [60]. Given a pathogenic role for IgM, B-cell depletion (e.g., rituximab) seems a reasonable therapeutic option in positive cases.

## 8. C1q Nephropathy

First described in 1985 in older children and young adults with proteinuria and hematuria, C1qN is characterized by bright C1q immunofluorescence with variable LM, including MCD, MesPGN, proliferative glomerulonephritis, and FSGS (see Table 3) [61]. However, on IFM, a “full-house” pattern of positive staining (IgG, IgM, IgA, C3, C1q) was found, mimicking systemic lupus erythematosus (SLE) nephritis with the only difference being the lack of endothelial cell tubuloreticular inclusions (TRIs) on EM; EDDs were predominantly mesangial/paramesangial with scant capillary wall deposits. No patient had symptoms/signs/serologies supporting SLE, and complement levels were normal. The biopsies were not consistent with immune complex-membranoproliferative glomerulonephritis (IC-MPGN), the other glomerulopathy likely to stain for C1q. The response to steroids was found to be poor. Iskandar et al. reported 15 pediatric cases with nearly identical clinical and pathologic features [62].

In 2003, Markowitz et al. found 19 (0.21% of biopsies) children and adults with C1qN defined as dominant/co-dominant mesangial C1q staining, mesangial EDDs, and no evidence for SLE [63]. IgG was present in all cases but only 4/19 (21%) had “full-house” positivity. About 80% had nephrotic-range proteinuria and 50% had INS; 22% had hematuria. By LM 17/19 had FSGS, 1 MCD, and 1 MesPGN. Response to steroids was poor. Hence, the authors posited that C1qN belongs in the primary podocytopathy disease spectrum.

Sharman et al. identified nine British patients initially considered to have seronegative lupus nephritis otherwise consistent with C1qN [64]. All nine had “full-house” positivity with negative SLE serology. All nine had mesangial deposits, but unlike other cases of C1qN, all nine had subendothelial and three had subepithelial EDDs; however, none had the endothelial TRIs typical of lupus nephritis and none developed SLE clinically or serologically after median 6-year follow-up. There was no consistent response to immunosuppression.

Fukuma et al. reviewed 30 Japanese children with C1qN, including 18 with asymptomatic urinary sediment abnormalities detected on routine screening and 12 with INS [65]. By LM, 22 had MCD, 6 had MesPGN, and 2 had FSGS. All 12 with INS received prednisone +/− cyclosporine; at follow-up, 3 were in remission, 8 were frequent relapsers, and 1 reached ESKD. Of the asymptomatic 18, 8 were normal, 9 had persisting urinary sediment abnormalities, and 1 reached ESKD.

Expanding on this work, Hisano et al. added 31 more patients to give 61 total Japanese C1qN patients defined as mesangial C1q ≥ 2+ by IFM, mesangial/paramesangial EDDs on EM (required), and lack of clinical/serologic evidence of SLE [66]. The 61 were divided into 36 with asymptomatic urinary sediment abnormalities and 25 with INS. By LM, 46 had MCD, 7 had MesPGN, and 8 had FSGS. On IFM, 38/61 (62%) were IgG+, 10/61 were IgM+ (16.4%), and 33/61 (54%) were C3+. All 25 with INS received prednisone +/− cyclosporine; at follow-up, 8 patients had normal urinalyses, but 13 were frequent relapsers. Of 8 patients with repeat biopsies, 3 lost C1q deposits, but 2 of these 3 showed segmental sclerosis. One of 25 with INS reached ESKD as did 1/36 with sediment abnormalities.

**Table 3:** **Selected series of C1q nephropathy.**

Author	Number	Clinical Features	Light Microscopy	IFM	Response	Follow-Up	Comments
Jennette/1985 [61]	15 Ages: 14–27	Proteinuria 100% NRP: 10 Hematuria: 6 Creat: < 1.5 mg/dL	MCD: 2 MesPGN: 3 Proliferative GN: 8 * Inadequate: 2	C3: 100% IgG: 100% IgM: 100% IgA: 60%	Steroids: 9 No response	Limited to 11: mean 7 months	First description
Iskandar/1991 [62]	15 Ages: 2–16	Proteinuria: 100% NS: 10 Hematuria: 5 C_creat_ < 60: 9	MCD: 8 FSGS: 7	C3: 14/15 IgG: 100% IgM: 100% IgA: 100%	NS: 5/9 unresponsive to prednisone Bolus steroids: MCD: 3 of 4 had remission	Unclear duration	Full-house IFM 1 reached ESKD
Markowitz/2003 [63]	19 Ages: 3–42	Proteinuria: 100% NRP: 15 (79%) NS: 9/18 (50%) Hematuria: 22% Creat > 1.2: 5 (28%)	MCD: 2 FSGS: 17 (including 6 collapsing and 2 cellular)	C3: 52.6% IgG: 100% IgM: 84% IgA: 32%	12 treated: 1 complete and 6 partial remissions 4 progressed (all FSGS), 2 reached ESKD	Available for 16/19: mean 27 months	FPE: 20–100% (mean 51%)
Levart/2005 [67]	12 Ages: 4–16	Proteinuria: 100% NS: 8 (75%) Renal insufficiency: 2	MCD: 4 MesPGN: 2 FSGS: 6	C3: 11/12 IgM: 100% IgG: 9/12 IgA: 7/12	1 MCD: remission 3 MCD: dependent 3 FSGS: resistant 1 FSGS: dependent	Range 0.5–17 years	Full-house: 7/12 FPE: 8/9 with NRP/NS EDDs in 9: including capillary wall
Fukuma/2006 [65]	30 Ages: 3–15	Asympt: 18 NS: 12 Hematuria: 16/18 asymptomatic and 1/12 with NS	MCD: 11/18 asympt 11/12 NS MesPGN: 6/18 asympt FSGS: 1/18 asympt 1/12 NS	C3: 17/30 IgG: 20/30 IgM: 5/30 IgA: 0/30	All 12 with NS treated (12 given prednisone, 3 CysA): 3 remission, 1 HD, 8/12 frequent relapsers	Means: asymptomatic: 6.9 years; nephrotic: 4.8 years	8/18 asympt normal at end, 9/18 remained asympt, 1 ESKD
Sharman/2004 [64]	9 Ages 19–63	Proteinuria: 100% NS: 1/9 Hematuria: 8/9	MesPGN: 100% Crescents: 1/9	C3: 100% IgG: 100% IgM: 100% IgA: 100%	Prednisone +/− C/A: 4 patients	Median 6 years	All 9 initially considered seronegative lupus nephritis
Vizjak/2008 [68]	72 Ages 2–66	Proteinuria: 61/72 NRP/NS: 34/72 Hematuria: 50/72 Reduced GFR: 33/72	MCD: 17 MesPGN: 20 FSGS: 11 Other: 14	C3: 83.3% IgG: 66.7% IgM: 80.6% IgA: 47.2%	Prednisone +/− alkylating agents or cyclosporine	Mean 6 years	Of 13 with INS and MCD, 10 had complete and 3 partial remissions. Of 9 with FSGS, 3 complete and 2 partial remissions
Hisano/2008 [66]	61 Ages 1–67	Asympt: 36 INS:25	MCD: 46 MesPGN: 7 FSGS: 8	C3: 54% IgG: 62% IgM: 16.4%	Prednisone: all INS and 9/36 asympt Cyclosporine in 6 INS INS: 8/25 normal, 13 FRs Asympt: 1/9 normal	Mean 7.2 years	On re-biopsy, 3 lost C1q, but 2 of these 3 progressed to FSGS
Wong/2009 [69]	9 Ages 1.3–15	INS 100% Hematuria: 43%	MCD: 100%	C3: 44% IgG: 100% IgM: 78%	Prednisone: 100% initially: 4/9 steroid sensitive 3/9 steroid-dependent 2/9 steroid-resistant	Median 64 months	Compared with MCD: more likely CNIs or MMF 100% eventually attained remission
Gunasekara/2014 [70]	35 Ages 0.5–16	Proteinuria +/− hematuria: 4 INS: 31	MCD 19 MesPGN: 3 FSGS/FGGS: 13	N/A	Prednisone 24/25 ** 12/25 s line drug(s) Complete remission: 11/13 MCD, 2/9 FSGS/FGGS, 0/3 MesPGN	Mean 4.1 years in 25 with >1 year follow-up	C1qN-MCD compared with non-C1qN-MCD: more relapses

* It is unclear if any or all of these had mesangial proliferation alone or focal or diffuse endocapillary proliferation. ** 25 had 1 year of follow-up. Asympt: asymptomatic urinary sediment abnormalities; C_creat_: creatinine clearance; C/A: cyclophosphamide or azathioprine; CNI: calcineurin inhibitor; Creat: serum creatinine; FGGS: focal global glomerulosclerosis; FPE: foot process effacement; FR: frequent relapse; GN: glomerulonephritis; IFM: immunofluorescence microscopy; MMF: mycophenolate mofetil; NRP: nephrotic range proteinuria; NS: nephrotic syndrome.

Kersnik Levart et al. described 12 pediatric C1qN patients from Slovenia, including 6 FSGS, 4 MCD, and 2 MesPGN [67]. Eight presented with INS and were treated with steroids: 1 responded, 4 became steroid-dependent, and 3 were steroid-resistant. Expanding on this work, Vizjak et al. evaluated 72 patients from Slovenia, 28 children (including the 12 described above) and 54 adults, with C1qN defined as dominant or co-dominant mesangial C1q ≥ 2+ without evidence for SLE or IC-MPGN [68]. LM revealed MCD 27, MesPGN 20, FSGS 11, or various other lesions. Clinically, 33 presented with NRP/NS, including 17/27 with MCD, 5/20 with MesPGN, and all 11 with FSGS. Others presented with non-nephrotic proteinuria or hematuria. Other reactants included IgG (66.7%), IgM (80.6%), IgA (47%), and C3 (83%); “full-house” was found in 22 (30.6%) predominantly in those with MesPGN by LM. Mesangial EDDs were found in 48 of 53, with 14 also having capillary wall deposits; FPE was ≥50% in half of 16 MCD with INS, 12% of 17 MesPGN with INS, and 71% of 7 FSGS with INS. Repeat biopsies revealed progression from MCD to MesPGN in two cases and to FSGS in one case. They noted two main clinicopathologic subsets: one with INS (a frequently relapsing course) and a podocytopathy (MCD or FSGS) by LM, and the other an immune-complex type of glomerulopathy with urinary sediment abnormalities and/or reduced GFR having either MCD or MesPGN by LM.

Wong et al. studied nine C1qN children from New Mexico with INS [69]. All had MCD by LM. IgG was positive in 100%, IgM 78%, C3 44%. All nine eventually achieved remission; four were initially steroid-sensitive, three steroid-dependent, two steroid-resistant. All eventually received calcineurin inhibition or mycophenolate. Compared with non-C1qN-MCD patients, C1qN-MCD patients were more frequently relapsing and steroid-resistant.

Gunasekara et al. followed 35 Sri Lankan children with C1qN, including 19 with MCD, 3 with MesPGN, and 13 with FSGS/focal global glomerulosclerosis (FGGS) [70]. Thirty-one presented with INS and 4 with proteinuria +/− hematuria. Besides C1q, 27 stained positive for IgM, but other immunoglobulins and complement staining were not reported. Of 13 with MCD treated with steroids having 1 year of follow-up, 11 had complete remission. Compared with C1q-negative MCD patients, the C1q-positive group had more and earlier relapses but otherwise no difference during follow-up. Additionally, 2/9 treated FSGS/FGGS patients followed for 1 year had complete remission, as did 0/3 with MesPGN.

Peng et al. evaluated 389 Chinese children with primary FSGS and found 18 (4.63%) had C1qN [71]; 10/18 were C3+, 4/18 IgG+, 17/18 IgM+, and 7/18 IgA+ (2/18 were “full-house” positive). All 18 had mesangial EDDs, and 2 had subendothelial deposits, one of which also had subepithelial deposits. Clinically, 7/18 had INS, 13/18 had hematuria, and 5/18 had hypertension. All 18 with INS received steroids and 17 received other immunosuppressive agents. Initially, 5 were steroid-sensitive and 13 steroid-resistant. Compared with 18 age, sex, and period of biopsy-matched C1q-negative primary FSGS patients, there was no significant difference in long-term remission, ESKD, or the combined end-point (≥40% decline in eGFR or ESKD), suggesting that, with segmental sclerosis, C1q-positivity did not matter like IgM-positivity.

Kim et al. evaluated 23 adult Korean patients with C1qN defined as ≥2+ mesangial C1q-positivity and mesangial/para-mesangial EDDs and compared them to 23 MCD and 23 FSGS patients matched for age, sex, and period-of-biopsy [72]. Of the 23 C1q-positive patients, 3 had MCD, 1 had increased mesangial matrix, 8 had MesPGN, and 11 had FSGS, including 8/23 with UPCR > 3.0. Four developed ESKD. The presence of mesangial hypercellularity, segmental sclerosis, or the degree of FPE was not related to ESKD within the C1q-positive group. However, comparing the C1q-positive group as a whole to the FSGS control group, ESKD was not significantly different (17.4% versus 30.4%, *p* = 0.491); no MCD controls reached ESKD.

To summarize, C1qN is characterized by significant (≥2+) mesangial C1q staining. Other immunoglobulins (IgM, IgG, and/or IgA) are usually present and may stain at the same, lower, or even higher intensity than C1q. A significant minority will have “full-house” positivity (IgG, IgM, IgA, C1q, C3) resembling lupus nephritis; however, TRIs are absent, patients are serologically unremarkable, and SLE does not develop over time. IC-MPGN must also be excluded. Like IgMN, the LM of C1qN is variable and includes MCD, MesPGN, and FSGS. In the absence of segmental sclerosis, C1qN has a prognosis worse than C1q-negative MCD; however, C1qN with segmental sclerosis is probably not worse than non-C1qN FSGS.

## 9. Pathogenesis of Primary Mesangial Proliferative Glomerulonephritis

In patients presenting with hematuria and/or non-nephrotic proteinuria, the mesangium is the likely primary site of disease, especially if the biopsy is consistent with IgMN or C1qN. In the setting of INS with obvious podocyte injury (diffuse FPE) plus mesangial proliferation, however, two main possibilities exist for the initiation of disease: a primary podocytopathy with secondary mesangial activation versus a primary mesangial insult with secondary podocyte dysfunction.

Some authors hypothesize that mesangial proliferation represents a transition state from MCD to FSGS [12,73]. In this scenario, the primary site of disease is the podocyte, with mesangial cells secondarily involved. In our opinion, the primary podocytopathies, MCD and FSGS, represent neither two separate entities nor the continuum of a single entity. The diffuse podocyte injury may be the final common phenotype resulting from one or more circulating substances that are toxic to podocytes and/or autoantibodies that target podocyte-expressed proteins, most notably nephrin, but including others, enhanced by a susceptible genetic background. We recently reviewed in detail these potential circulating factors and autoantibodies [74]. In this scenario, mesangial cells would be secondarily activated to proliferate and produce increased matrix in response to podocyte injury, justifying classification of MesPGN with primary podocytopathies. This would best apply to patients with INS having diffuse FPE and lacking immune complexes on IFM and EDDs on EM.

**Figure 1:**
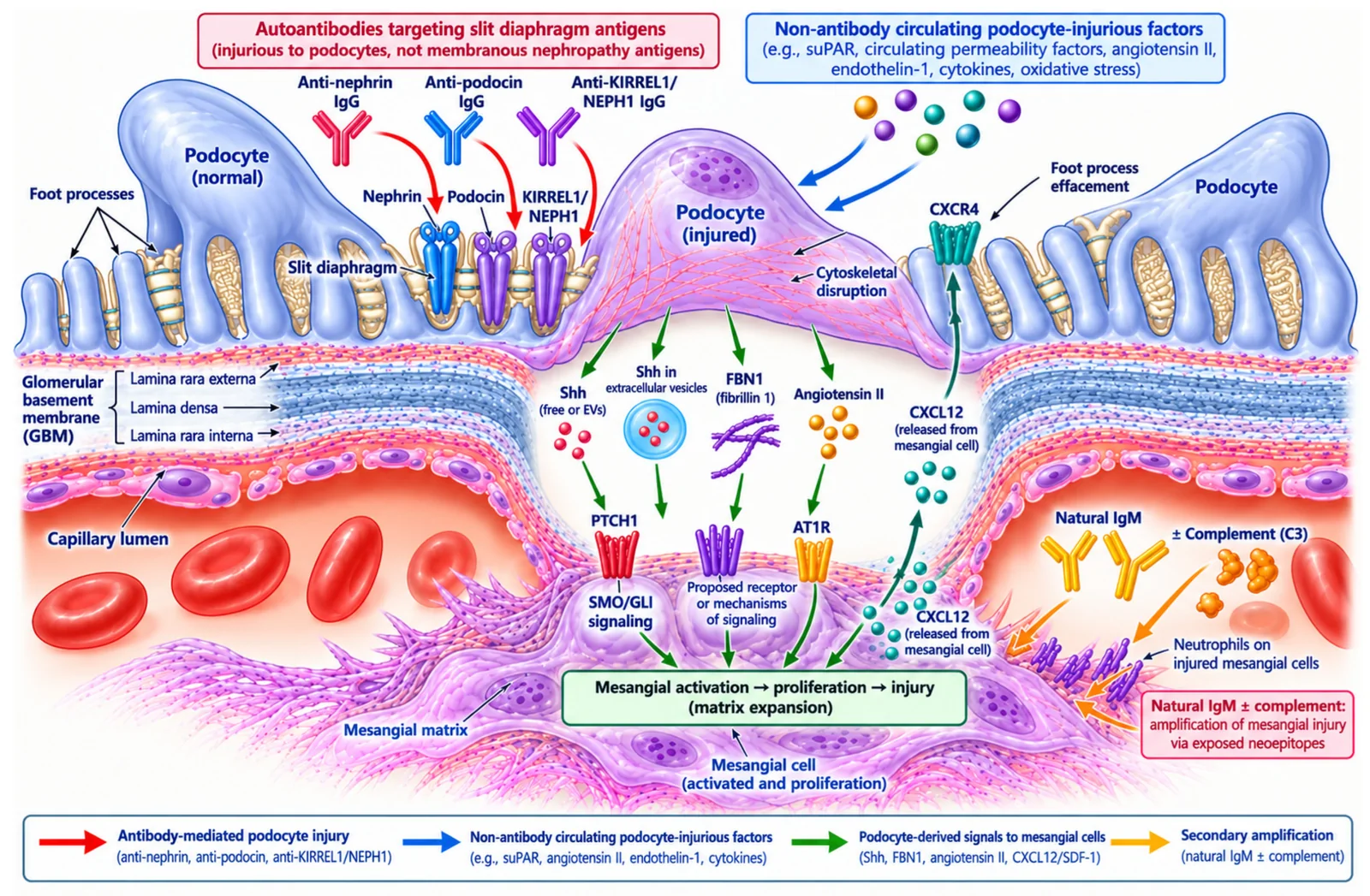
Primary podocyte injury resulting in secondary mesangial cell activation and proliferation. Upon being injured by antibodies or other circulating factors, podocytes release substances that then activate mesangial cells to proliferate, including Shh (sonic hedgehog) which may circulate freely or as bound to extracellular vesicles, FBNI (fibrillin 1), angiotensin II, or CXCR4, each of which may bind to their respective receptors expressed on mesangial cells and induce activation, proliferation, and injury. Natural IgM +/− complement may be involved as an amplifier (as opposed to an initiator) of injury reacting to neoepitopes exposed by mesangial cell injury.

It is well known that cross-talk between podocytes and mesangial cells exists and may explain how mesangial cells get secondarily activated in primary podocytopathies (see Figure 1) [75]. Zhou et al. demonstrated that podocyte sonic hedgehog (Shh) is upregulated in animal models of chronic kidney disease and selectively induces mesangial cell activation and proliferation in vitro and in vivo [76]. Subsequently, Liu et al. demonstrated increased production of extracellular vesicles (EVs) rich in Shh in injured mouse podocytes that induced activation and proliferation of mouse mesangial cells in vitro [77]. Wu et al. found that expression of the C-X-C chemokine receptor type 4 (CXCR4) and angiotensin II were increased and colocalized in injured podocytes along with upregulation of their respective receptors (stromal cell-derived factor 1α and the type-1 receptor of angiotensin II) in mesangial cells, highlighting another mechanism of podocyte–mesangial cell cross-talk [78].

Most recently, He et al. demonstrated podocyte upregulation of fibrillin-1 (FBN1) both in patients with proteinuric kidney disease and animal models of glomerular disease. Mesangial cells were activated and proliferated in response to FBN1 both in vitro and in glomerular mini-organ cultures [79]. Podocyte-specific knockout of the FBN1 gene reduced mesangial cell activation and glomerulosclerosis in an animal model of FSGS. Hence, mechanisms are being unraveled as to how primary podocyte injury can lead to mesangial injury/proliferation, matrix production, and eventual glomerulosclerosis, supporting the classification of MesPGN with the primary podocytopathies. Although this scenario would apply most to MesPGN lacking immune complexes, IgM deposits may represent natural IgM antibodies reacting with injured mesangial cells functioning as secondary amplifiers and not the initiators of the process.

**Figure 2:**
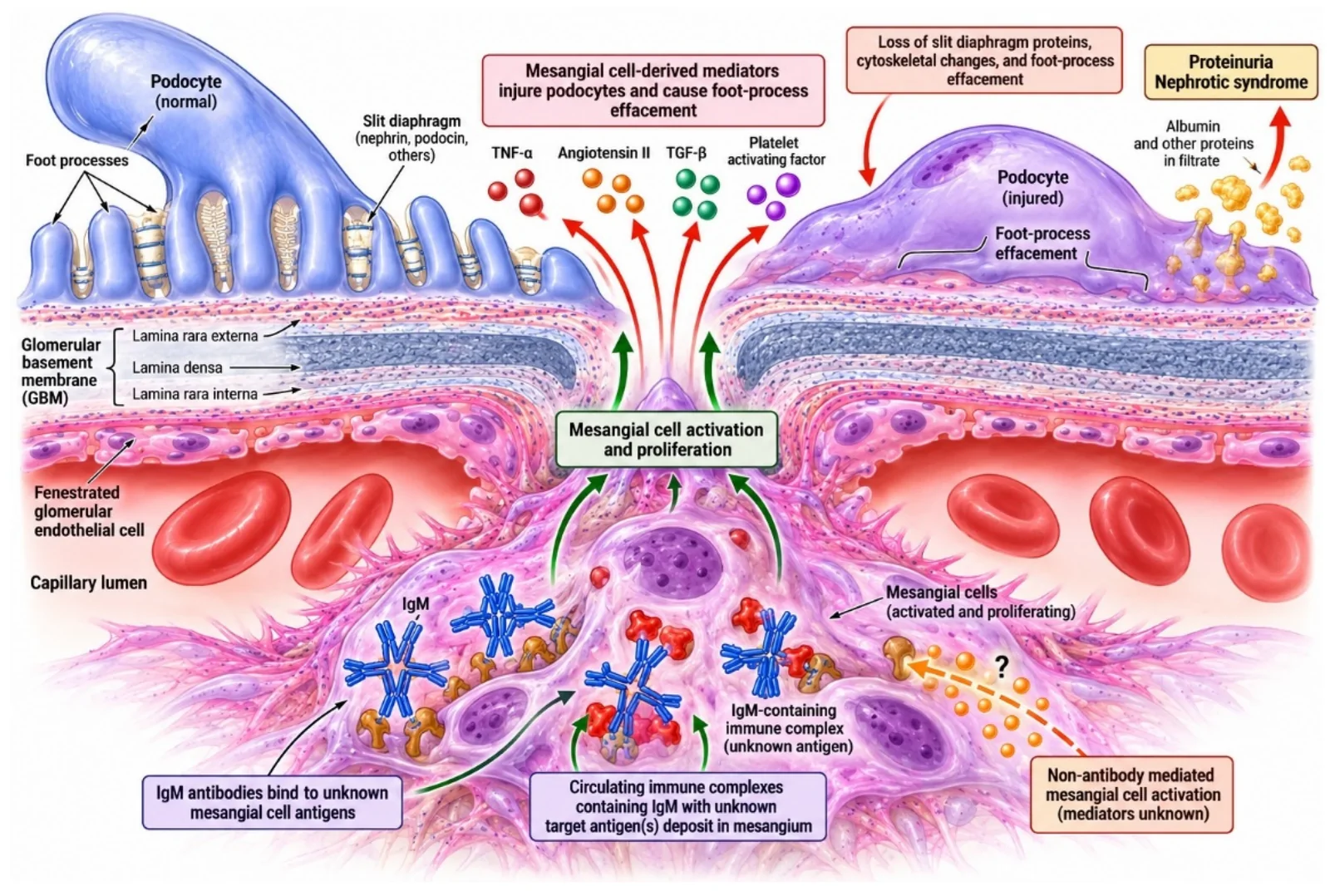
Primary mesangial cell injury with mesangial cell–podocyte cross-talk. IgM antibodies may react with unknown mesangial cell antigen(s), or IgM-containing immune complexes composed of IgM and unknown antigen(s) may form in the circulation and deposit in the mesangium. Either way, mesangial cells get activated, proliferate, and release substances injurious to podocytes, resulting in foot process effacement, proteinuria, and nephrotic syndrome. Potential substances include TNF-α, angiotensin II, TGF-β, and platelet-activating factor. Note that mesangial cells may also get activated by unknown non-antibody-mediated factors.

By contrast, MesPGN could result from primary mesangial cell injury with secondary disruption of podocyte function (see Figure 2). IgM deposits +/− complement (IgMN) or C1qN would best fit this scenario, especially if only segmental FPE. Cross-talk between mesangial cells and podocytes is well described even in common kidney diseases such as diabetes [80] and hypertension [81].

Patients with two other immune-complex diseases (IgAN and lupus nephritis) that result in mesangial proliferation with mesangial immune complexes may clinically develop florid nephrotic syndrome with diffuse FPE in the absence of capillary wall deposits. A significant minority of patients with IgAN present as INS, and on biopsy they can have normal glomeruli or mild mesangial proliferation but diffuse FPE [2]. IgA deposits and EDDs are restricted to the mesangium. Clinically, besides explosive onset of nephrotic syndrome, these cases usually have normal GFR, normotension, and marked steroid sensitivity, mimicking the typical MCD patient.

These cases could represent the chance occurrence of two glomerulopathies (MCD and IgAN) [2]. Additionally, the patient may simply have MCD with lanthanic IgA deposits, as may occur in the general population. Most relevant to this discussion, the disease may simply be IgAN with pronounced podocyte injury due to mesangial–podocyte cross-talk. Cytokines produced by activated mesangial cells in IgAN may directly activate podocytes [82], including TNF-α, angiotensin II, TGF-β, and platelet-activating factor, and one could postulate the same occurs in MesPGN.

Furthermore, the pathogenesis of IgAN involves deposition of galactose-deficient IgA1-containing immune complexes in the mesangium with mesangial cell activation, analogous to what may occur in IgMN, although the antigenic target in IgMN is undefined. A prime determinant of prognosis in IgAN is the degree of proteinuria, heralding secondary podocyte injury and dysfunction [83], and directly correlating with the degree of podocyte FPE [84]. Effacement is the initial adaptive response of podocytes to insult. With sustained injury, podocyte hypertrophy and then depletion may occur, fostering adhesions to Bowman’s capsule. The same may occur in IgMN, making the not uncommon finding of segmental sclerosis on repeat biopsies of IgMN analogous to the S lesion of the IgAN Oxford classification.

Lupus podocytopathy is an uncommon form of lupus nephritis (~1%) characterized clinically by nephrotic syndrome usually in the setting of active SLE, a steroid-sensitive but relapsing course, and susceptibility to AKI [85,86,87]. Pathologically, minimal changes, mesangial proliferation, or segmental sclerosis may be found with or without mesangial immune complexes. The distinguishing feature of lupus podocytopathy is diffuse FPE (>70%) without the capillary wall deposits (subendothelial or subepithelial) that occur with proliferative and membranous variants of lupus nephritis.

A recent systematic review of lupus podocytopathy found 26 studies involving 259 patients, of which 113 had minimal changes, 91 mesangial proliferation, and 43 FSGS [87]. The vast majority had NRP and most presented at the time of initial SLE presentation. The vast majority responded to steroids, although FSGS cases were less responsive (84.6% vs. 98.9% of MCD cases); 20 eventually developed classic lupus nephritis and 3 reached ESKD.

Hence, both experimental and clinical studies support the possibility that primary mesangial disease, mediated by immune complexes or other mechanisms of mesangial cell activation/injury, may result in secondary podocyte injury presenting as INS with FPE in glomerular capillary loops. We suspect that at least some cases of MesPGN presenting as INS are mediated by this mechanism, especially those with positive mesangial immune complexes (IgMN, C1qN).

Overall, data are accumulating regarding mesangial cell–podocyte cross-talk both experimentally and clinically. Various mediators may be involved, as outlined above, and cross-talk can proceed in either direction. Whether and to what extent such cross-talk underlies the pathophysiology of this group of diseases (MesPGN, IgMN, C1qN) remains to be unraveled. We suspect significant heterogeneity exists between specific histologic patterns and from patient to patient.

## 10. Treatment of Primary Mesangial Proliferative Glomerulonephritis

Supportive care is indicated for all patients, including reducing overweight/obesity, exercise, smoking cessation, sodium restriction, strict BP control, RAS inhibition, and SGLT2 inhibition. Sparsentan, a dual blocker of the angiotensin II-type-1 receptor and the endothelin type-A receptor, was recently shown to significantly reduce proteinuria in patients with primary FSGS, although no significant effect on eGFR decline was demonstrated [88]; sparsentan has recently received FDA approval for FSGS without nephrotic syndrome.

The use of immunosuppression is guided by the clinical presentation. In patients with non-nephrotic proteinuria and/or hematuria, immunosuppression is not indicated. In cases with persisting proteinuria and eGFR declining for no other reason, especially in a child or young adult, immunosuppression may be considered.

## 11. Initial Immunosuppression

In patients with INS, in our opinion immunosuppression is indicated regardless of LM, IFM, or EM. There are no RCTs addressing immunosuppression for MesPGN, IgMN, or C1qN. At best, the level of evidence would be 4, i.e., based on case series and reports. Furthermore, some recommendations we make are level 5 (expert opinion only), as the benefit for MesPGN was assumed to be similar to that shown for a particular therapy in patients with primary podocytopathies (MCD and/or FSGS) and not specifically MesPGN. The 2021 KDIGO guidelines for the treatment of glomerular disease do not include sections on any of the three (MesPGN, IgMN, or C1qN) [8]. Herein we consider treatment recommendations for all three under the rubric MesPGN unless otherwise specified. Given the relationship to the primary podocytopathies, initial treatment in our opinion should be like that recommended by KDIGO for the initial treatment of MCD/FSGS, i.e., high-dose prednisone (1 mg/kg/day for 12–16 weeks, with tapering if responsive). Several RCTs compared alternative initial regimens to high-dose steroids for the primary podocytopathies (Table 4). None specifically targeted MesPGN, but we suspect that trials of MCD and/or FSGS included patients with mesangial proliferation. Some studies have indeed separated MesPGN from MCD and FSGS without reporting differences in response rates [89,90,91] (vide infra), but most have not made this distinction.

Li et al. randomized 119 adult Chinese MCD patients to tacrolimus monotherapy versus high-dose oral prednisone, with both groups initially receiving 10 days of IV methylprednisolone, and found tacrolimus to be non-inferior, with no difference in remission at 12 weeks, time to remission, or relapse rate [92]. Medjeral-Thomas et al. randomized 50 adult British MCD patients to tacrolimus monotherapy or high-dose prednisone and found no difference in complete remission at 8 weeks, 16 weeks, or 26 weeks; there was no significant difference in relapse rate or serum creatinine over time [93]. Chin et al. randomized 144 adult Chinese MCD patients to tacrolimus plus low-dose prednisone (0.5 mg/kg) or the standard high-dose prednisone regimen and found no difference in complete remission by 8 weeks or time to remission but significantly fewer relapses with maintenance tacrolimus [94]. Hence, in our opinion a tacrolimus-based regimen is an alternative for the initial therapy of adults with MesPGN wishing to avoid high-dose steroid exposure. However, it remains uncertain how much concurrent steroid exposure, if any, is required.

Remy et al. randomized 116 French adults with MCD to mycophenolate sodium and low-dose prednisone or the high-dose prednisone regimen with no significant difference in complete remission at 4 weeks, 8 weeks, or 24 weeks [95]. The INTENT investigators randomized 269 German children, with their initial episode of INS in remission within 28 days of high-dose steroids, to either MMF for 12 weeks plus alternate-day prednisone over the first 2 weeks versus full-dose prednisone for 6 weeks plus alternate-day prednisone for another 6 weeks [96]. The primary endpoint, treated relapses within the 24 months following the initial 12-week treatment, occurred in 79.1% of the MMF group versus 74.8% of control, showing statistical non-inferiority for MMF with significantly fewer steroid-related side-effects.

Kristensen et al. randomized 67 Danish adult MCD patients to active vitamin D (alfacalcidol) plus low-dose prednisone or high-dose prednisone and found no difference in remission at 16 weeks, with significantly fewer adverse events and no difference in relapse rates [97].

Although no RCTs assessed rituximab as initial therapy for podocytopathies, observational data from three small retrospective studies totaling 25 adult MCD patients showed a benefit [98,99,100]. Li et al. performed a prospective cohort study in 74 adult Chinese MCD patients comparing rituximab 1 g twice in 20 patients, half-dose prednisone (0.5 mg/kg) plus rituximab 1 g once in 28, and full-dose prednisone in 26 as initial therapy and found complete remissions in 50%, 96%, and 96%, respectively, at 12 months with reduced steroid-induced diabetes in the half-dose steroid regimen [101]. Rituximab alone appeared less effective. By contrast, Zhao et al. compared rituximab (four weekly doses of 375 mg/m^2^) to standard full-dose prednisone in 82 adult Chinese MCD/FSGS patients, including nine MCD patients receiving rituximab as initial therapy [102]. All nine attained remissions, including eight complete.

**Table 4:** **Randomized controlled trials for initial treatment of primary podocytopathies potentially applicable to mesangial proliferative glomerulonephritis presenting with nephrotic syndrome.**

Trial	Age	Number	Histology	Treatment	Primary Endpoint	Results	Comment
Li [92] 2017	A	119	MCD	IV methylprednisolone for all Tacrolimus versus steroids	Total remission (CR or PR)	98.3% versus 96.2%, *p* = NS	Similar CR rate and relapse rate
Medjeral-Thomas [93] 2020	A	50	MCD	Tacrolimus versus steroids	CR at 8 weeks	68% versus 84% *p* = 0.32	No difference in relapse rate
Chin [94] 2021	A	144	MCD	Tacrolimus plus half-dose steroids to full-dose steroids	CR within 8 weeks	79.1% versus 76.8%	Tacrolimus non-inferior; fewer relapses while on tacrolimus
Remy [95] 2018	A	116	MCD	MPA (720 mg twice daily) plus half-dose steroids versus full-dose steroids	CR within 4 weeks	64.9% versus 57.9%, *p* = 0.44	Similar remission rate at 8 and 24 weeks; no difference in relapse rate
Kristensen [97] 2026	A	67	MCD	Alfacalcidol plus half-dose steroids versus full-dose steroids	Remission rate	88% versus 91%	Non-inferiority not established; relapses: 35% versus 32%
Benz [96] 2026	P	269	NA All with INS in remission	12 weeks total MMF: 600 mg/m^2^ twice/day versus prednisone 60 mg/m^2^/day tapered	Treated relapse within 24 months of completing treatment; non-inferiority	79.1% vs. 74.8%	MMF non-inferior with reduced steroid toxicity

A: adults; CR: complete remission; MMF: mycophenolate mofetil; MPA: mycophenolate sodium; NA: not available; P: pediatric; PR: partial remission.

Wang et al. compared 14 Chinese MCD patients (12 adults) treated with rituximab (various dosages) +/− short-course high-dose prednisone to 14 propensity-matched patients treated with the high-dose regimen and found 100% remissions overall (11 complete remissions in each group) at 24 weeks; 5 rituximab-treated patients with a year or more of follow-up had no relapses, although 3 other patients relapsed after 24 weeks [103].

The results using rituximab as initial therapy for FSGS are less encouraging, although there are minimal data. Roccatello et al. gave eight adult primary FSGS patients high-dose rituximab (eight weekly infusions of 375 mg/m^2^) and attained only a partial remission in one patient [104].

## 12. Frequently Relapsing or Steroid-Dependent INS

Such patients are steroid-sensitive, so the presence or absence of segmental sclerosis does not change the approach. For children with frequently relapsing/steroid-dependent (FR/SD) INS, KDIGO 2025 guidelines for children recommend one of five agents: CNIs, cyclophosphamide, levamisole, mycophenolate mofetil (MMF), and rituximab in no special order [105]. For adults with FR/SD MCD, the KDIGO 2021 guidelines recommend cyclophosphamide, rituximab, CNIs, or mycophenolic acid analogs as steroid-sparing agents, without preferring one class [8]. For FR/SD FSGS, the recommendations would be the same.

No trials specifically targeted MesPGN, although several RCTs addressed steroid-sparing agents in FR/SD INS in general (see Table 5). Sinha et al. randomized 80 Indian children with FR/SD INS upon attaining remission to oral prednisolone (0.5–0.7 mg/kg) on alternate days or daily during infection versus oral levamisole on alternate days for 1 year and found frequent relapses in 40% with prednisolone and 22.5% with levamisole (*p* = 0.0091); prednisolone was not non-inferior [106]. However, the proportion of patients in sustained remission was similar, as was the incidence density of relapses.

Wang et al. randomized 270 Chinese children/adolescents with FR/SD INS in remission to tacrolimus or MMF for 1 year and found tacrolimus was superior at preventing relapse (82% versus 58.5%, *p* < 0.001) and showed a significantly lower cumulative steroid dosage [107].

Rituximab has been studied in several RCTs and numerous observational studies for FR/SD INS in both children and adults. Various dosing regimens have been used. High-dose regimens are approximately 1500 mg/m^2^ (four weekly doses of 375 mg/m^2^ or 1 g twice, 15 days apart). Moderate dosing is approximately 750 mg/m^2^ (two 375 mg/m^2^ weekly doses or a single 1 g or 750 mg/m^2^ dose). Low-dose rituximab is a single 375 m/m^2^ dose. A very low dose of 100 mg/m^2^ was used but associated with a significant increase in relapse rate [108]. Although a minority of patients with FR/SD INS receiving rituximab will not relapse without subsequent rituximab or other immunosuppression [109], the majority will. Factors affecting relapse rate include the initial dosage schedule, concurrent maintenance immunosuppression, or repeated rituximab dosing.

**Table 5:** **Randomized controlled trials for treatment of frequently relapsing/steroid-dependent primary podocytopathies potentially applicable to mesangial proliferative glomerulonephritis with nephrotic syndrome.**

Trial	Age	Number	Histology	Treatment	Primary Endpoint	Results	Comment
Sinha 2024 [106]	P	160	INS Biopsy N/A	Levamisole (2–2.5 mg/kg on alternate days) versus prednisolone (0.5–0.7 mg/kg on alternate days)	Proportion with frequent relapses	22.5% versus 40% Prednisone was not non-inferior	Similar proportion in sustained remission, relapses comparable
Wang 2025 [107]	P	270	INS Biopsy N/A	Tacrolimus versus MMF	1-year relapse free survival	Relapse-free survival significantly greater with tacrolimus (HR, 2.86, *p* < 0.001)	Tacrolimus group required less steroids
Basu 2018 [110]	P	120	MCD: 85 FSGS: 35	Tacrolimus plus tapering alternate day steroids versus rituximab 375 mg/m^2^ twice	12-month relapse-free survival	63% versus 90%, *p* < 0.001 in favor of rituximab	Lower steroid exposure with rituximab
Iijima 2014 [111]	P	48	MCD: 44 FSGS: 3	Rituximab 375 mg/m^2^ weekly x4 versus placebo	Relapse-free period	267 days versus 101 days, *p* < 0.0001 in favor of rituximab	No significant difference in serious adverse events
Isaka 2025 [112]	A	72	MCD: 57 FSGS: 5	Rituximab 375 mg/m^2^ on weeks 1, 2, 25 versus placebo	Relapse-free at 49 weeks	87.4% versus 38%, *p* < 0.001 in favor of rituximab	Hazard ratio for relapse with rituximab 0.16, *p* < 0.001

FSGS: focal segmental glomerulosclerosis; HR: hazard ratio; INS: idiopathic nephrotic syndrome; MCD: minimal change disease; MMF: mycophenolate mofetil; N/A: not available; P: pediatric.

Basu et al. randomized 120 Indian children with SD INS (~70% MCD, 30% FSGS) in remission to moderate dose rituximab (all 60 received two doses of 375 mg/m^2^) or tacrolimus, with both groups receiving tapering alternate-day prednisolone. Rituximab had a significantly higher 12-month relapse-free survival (90% versus 63%, *p* < 0.001). The cumulative steroid dose was lower with rituximab (22.5 versus 86.3 mg/kg) [110].

Two placebo-controlled, double-blind RCTs from Japan showed the significant ability of rituximab to reduce the relapse rate. Iijima et al. randomized 48 children/adolescents (44 MCD, 3 FSGS, 1 unknown) with complicated (defined as receiving additional immunosuppressants plus steroids) FR/SD INS in remission to high-dose rituximab or placebo [111]. Patients also received prednisolone 60 mg/m^2^ on alternate days tapered over six weeks. The median relapse-free period was significantly longer with rituximab (267 days versus 101 days, *p* < 0.001), and serious adverse events were non-significantly less with rituximab (26% versus 42%). Isaka et al. randomized 66 adults with FR/SD INS (57 MCD, 5 FSGS, 4 other) in remission to moderate-dose rituximab (375 mg/m^2^ on weeks 1, 2 and 25) or placebo [112]. Steroids or other immunosuppressants were weaned similarly in both groups. Rituximab resulted in a significantly greater relapse-free survival at week 49 (87.4% versus 38%, *p* < 0.001).

Observational data support the use of rituximab for FR/SD INS. Ruggenenti et al. gave rituximab 375 mg/m^2^ (with a second dose if B-cells were still detectable at one week) to 30 patients (10 children) with SD/FR INS in remission, including 22 with MCD or MesPGN and 8 with FSGS, and found that all were in remission at 1 year, including 18 treatment-free, and 15 never relapsing [91]; MesPGN was not considered separately. Total relapses decreased from 88 the year before rituximab to 22 after, and the per-patient steroid median maintenance dosage decreased from 0.27 mg/kg to 0 mg/kg (*p* < 0.001). A recent systematic review and network meta-analysis found rituximab to be the most favorable agent for pediatric FR/SD INS, with the fewest relapses and resulting in the lowest steroid exposure [113].

Maxted et al. retrospectively evaluated 60 pediatric FD/SD INS patients comparing low-, medium-, and high-dose rituximab and found no difference in 12-month relapse rate between the various dosing strategies [114]. By contrast, Chan et al., in a multinational study, evaluated 511 children with FR/SD INS in remission of which 191, 208, and 112 were treated with low-, medium-, or high-dose rituximab, respectively [115]. Additionally, 283 (55%) received maintenance immunosuppression (corticosteroids, MMF, or CNIs). Relapse-free survival was significantly shorter with the low-dose regimen without maintenance immunosuppression. However, the low-dose regimen with maintenance immunosuppression had similar outcomes to the moderate and high-dose regimens, which were not significantly different from each other, with or without maintenance immunosuppression.

In a placebo-controlled, double-blind, pediatric RCT of Japanese patients with complicated FR/SD INS, 78 were initially given high-dose rituximab and then randomized to MMF or placebo for 505 days; no significant difference in treatment failure by end of follow-up (median 784 versus 472.5 days, *p* = 0.07) was found, although a post-hoc analysis showed significant benefit if analysis was restricted to time on study drug [116].

Ito et al. found MMF effective in reducing relapse in nine complicated pediatric SD patients following rituximab compared with historical controls receiving just rituximab [117]. In a non-randomized trial in 29 Japanese children with complicated SD INS given low-dose rituximab, Fujinaga et al. found cyclosporine more effective than MMF for maintaining remission following rituximab [118].

Maintenance rituximab dosing to prevent relapses in SD/FR INS in both children and adults has been studied. An international retrospective study evaluated 346 children with FR/SD INS receiving two or more courses of rituximab [119]. Relapse-free survival increased progressively with each subsequent course from 10 months to 12 to 16 months despite no difference in duration of B-cell depletion. Side-effects were considered acceptable (hypogammaglobulinemia in 50.9%, neutropenia 3.7%, infection 4.5%). Similarly, Sinha et al. found sequential rituximab doses effective at reducing relapses in a single-center study of 250 Indian children with complicated SD, steroid-resistant–CNI-dependent, or steroid/CNI-resistant INS and found a reduction of two relapses/person-year, a reduced prednisone dose, and withdrawal of additional agents in 62% [120]. Hypogammaglobulinemia occurred in 35%.

Several small studies in adults with MCD reported repeated low doses of rituximab (200 mg or 375 mg/m^2^) given at 6-month intervals effective at preventing relapses [121,122,123]. In an international retrospective study of adult patients (119 MCD, 64 FSGS) with difficult-to-treat INS (68% SD/FR, 22% steroid-resistant, 85% treated with ≥2 immunosuppressants) given rituximab at variable dosages, the RITERM Study Team found 151 (82%) in remission at 6 months, of which 83 (55%) achieved relapse-free survival at 36 months. Maintenance rituximab, given to 25%, significantly increased the chance of relapse-free survival with withdrawal of all immunosuppressive medications (61% versus 36%, *p* = 0.01) [124]. Similarly, a French, retrospective, multicenter study involving 134 adult patients with podocytopathies given rituximab found relapse frequency was significantly reduced and maintenance dosing between 6 and 12 months significantly reduced relapse risk (hazard ratio, 0.35, *p* = 0.02) [125].

Resistance to rituximab may occur for various reasons, including reduced bioavailability (urinary loss or internal uptake by B-cells) and development of anti-rituximab antibodies (ARAs). Rituximab redistributes CD20 into lipid rafts, enhancing complement-dependent cytotoxicity (CDC) [126]. Rituximab also promotes apoptosis [127] and antibody-dependent cellular cytotoxicity (ADCC) [128]. More potent B-cell depleters have been developed. Ofatumumab, a fully human type 1 anti-CD20 antibody, is more effective than rituximab in fixing complement as it binds to a different epitope, one closer to the cell membrane, facilitating better complement activation [129]. Obinutuzumab is a humanized type 2 anti-CD20 antibody with a glycoengineered Fc region having increased affinity for FcγRIIIa receptors that are expressed on effector cells (NK-cells, macrophages) markedly enhancing ADCC [130]. Rituximab and obinutuzumab recognize overlapping epitopes but bind in different orientations. As CD20 is not redistributed into lipid rafts, obinutuzumab does not effectively induce CDC [131]. Obinutuzumab also promotes non-apoptotic (lysosomal mediated) programmed cell death [132].

Up to one-third of patients with INS given rituximab may develop ARA [112,133], which may shorten B-cell depletion and/or promote earlier relapses or rituximab resistance. The alternative B-cell depleters ofatumumab and obinutuzumab appear to not cross-react with ARA and have been successfully used in ARA-positive patients with membranous nephropathy [134,135].

An RCT of 140 children and young adults with CNI- and steroid-dependent INS compared ofatumumab to low-dose rituximab and found no difference in 12- or 24-month relapse-free survival [136]. A small pediatric study of ofatumumab in multidrug-resistant INS was stopped for futility [137].

Several studies assessed obinutuzumab for INS. Dossier et al. retrospectively evaluated 41 rituximab-treated French children with FR/SD INS that did not have B-cell depletion (*n* = 8), had shortened B-cell depletion (<3 months, *n* = 6), or relapsed after B-cell recovery (*n* = 27) given low-dose obinutuzumab and obtained B-cell depletion in all lasting a median 8.3 months [138]. A similar median time of B-cell depletion occurred in the five with ARA (8.3 months). Remission occurred in 92% at 12 months and 68% at 24 months.

Parmentier et al. administered low-dose obinutuzumab to 32 pediatric ARA-positive French FR/SD INS patients that failed to B-cell deplete with rituximab (*n* = 9), had short B-cell depletion (*n* = 10), or relapsed, and found a median duration of B-cell depletion of 6.5 months with 62.5% relapse-free survival at 24 months; individual ARA concentrations generally declined over time [139]. Zha et al. obtained 100% remission at 6 months in 10 Chinese children with INS and rituximab failure (no remission or relapse within six months); B-cell depletion persisted throughout the six months [140].

Similar results were obtained in adults. Jin et al. treated six Chinese MCD patients with steroid- or multidrug-resistant MCD with obinutuzumab and obtained six complete remissions with no relapses after approximately one year [141]. Lin et al. gave obinutuzumab to 11 Chinese patients with primary podocytopathies (8 MCD, 3 FSGS) with no remission or quick relapse following prior rituximab with or without additional immunosuppression and obtained 10 complete remissions and 1 partial remission [142]. Li et al. gave obinutuzumab to 161 Chinese patients with SD/FR INS, including 53 with MCD, 28 with MesPGN, and 21 with FSGS [89]. After a median follow-up of 15 months, 148 (91.9%) achieved remission (98% for MCD, 92.9% for MesPGN, and 90.4% for FSGS), including 91 complete remissions. There were 17% relapses among remitters. These data suggest that MesPGN responds similarly to B-cell depletion as the primary podocytopathies, at least in those cases with a SD/FR course.

## 13. Steroid-Resistant INS

Steroid-resistance occurs in 10–30% of childhood INS and is typically defined as failure to remit completely with four weeks of high-dose steroids [8]. In adults with primary podocytopathies, the resistance rates are somewhat higher (30–40%), require longer courses to define (up to 16 weeks), and vary by histologic type (10–20% for MCD versus 25–65% for FSGS) [8]. Such resistance may occur at the outset of treatment, termed initial steroid resistance (ISR). By contrast, a patient may respond initially, but upon relapse become steroid-resistant, termed late steroid resistance (LSR). The pathophysiology may differ. Genetic defects underlie a significant minority of ISR cases, perhaps a third of pediatric cases [143], and ISR cases should undergo genetic testing to avoid unnecessary immunosuppression. Non-genetic ISR probably arises from immune dysregulation resulting in circulating factors or autoantibodies injurious to podocytes which may respond to alternative immunosuppression. Anti-nephrin antibodies were found in a minority of children with non-genetic steroid-resistance (14% in one study) and rarely in genetic steroid-resistance (2%) [144]. Ragliante et al. found anti-slit diaphragm IgG using high-resolution microscopy in 27% of ISR cases [145].

By contrast, LSR is not likely genetic, but strongly linked to circulating factors or autoantibodies. In the study of Ragliante et al. cited above, 87% of cases with LSR had anti-slit IgG antibodies [145]. Furthermore, in patients with steroid-resistant podocytopathy progressing to ESKD, post-transplantation recurrence is much more likely with LSR than ISR (92.9% versus 30.2% in one study, OR 30, *p* < 0.001), supporting the idea of a circulating factor or autoantibody in LSR [146].

Epigenetic mechanisms may be involved in steroid-resistance, including aberrations in DNA methylation, differential expression of micro-RNAs, and histone modifications [147]. It is unclear if such mechanisms are more likely involved with non-genetic ISR or LSR. Epigenetic modifications are known to modulate immune cell function and glucocorticoid receptor signaling and theoretically could accumulate over time and with repeated steroid courses, implicating a greater role in LSR. By contrast, a study found SOCS3 promoter hypomethylation in steroid-resistant INS versus steroid-sensitive cases before steroid exposure, and hence potentially contributing to ISR as well [148]. In the future, epigenetic modifications may be the target of personalized therapy, but much more work is required for this nascent field.

Steroid-resistant INS can also be subdivided into cases responding to intensified additional immunosuppression versus those that are multidrug-resistant. Importantly, neither steroid-resistance nor multidrug-resistance is defined by LM and may include cases with MCD.

Regarding treatment for steroid-resistance, KDIGO guidelines recommend CNIs as initial therapy without distinguishing ISR from LSR [8,105]. Several small RCTs involving children and adults with steroid-resistant INS showed a significant benefit for CNI compared with placebo/standard of care [149,150,151] or compared with intravenous cyclophosphamide [152,153]. Meta-analyses [154,155] support CNI as first-line therapy when steroid-resistant. A network meta-analysis of pediatric steroid-resistant INS found CNIs more efficacious than other agents (cyclophosphamide, MMF, leflunomide, chlorambucil); ISR and LSR were not separately analyzed [154]. A recent Cochrane review of RCTs for the treatment of pediatric steroid-resistant INS also found CNIs increased the likelihood of remission compared with either placebo/no treatment or cyclophosphamide [156]. No difference between ISR and LSR was evident, although subgroups were small.

In those wishing to avoid CNI toxicity, alternatives are available for steroid-resistant cases. The NIH-funded FSGS clinical trial found no difference in remission comparing pulse oral dexamethasone plus MMF to cyclosporin in 138 children and adults with primary FSGS [157]. Rituximab has also been studied for steroid- and multidrug-resistant INS. A small pediatric RCT comparing moderate-dose rituximab to standard-of-care involved 31 children with INS resistant to both steroids and CNIs for six months; there was no significant proteinuria reduction at three months [158]. Multiple very small observational studies assessed the ability of rituximab to induce remission in INS refractory to both steroids and CNIs and found overall remission rates of 18–80% (reviewed in [159]).

In the largest observational study to date, an international study of steroid-resistant pediatric INS involving 28 centers, Chan et al. retrospectively analyzed 246 non-remitted patients, including 146 with CNI-resistance (on CNIs for ≥6 months) and 100 with CNI administration for < 6 months (technically not yet CNI-resistant) [90]. Included were 112 with ISR and 134 with LSR. Histology included 136 with FSGS, 79 with MCD, 17 with MesPGN, 3 with IgMN, and 1 with C1qN. Overall remission rates at 3, 6, 12, and 24 months ranged from 26 to 39% in the CNI-resistant group and from 42 to 60% in the group on CNIs < 6 months; the outcome of the 17 with MesPGN was not reported separately. Both non-remission and partial remission were associated with worse kidney survival compared with complete remission.

Hence, rituximab appears effective in a subset of patients with steroid/CNI-resistant INS, at least in pediatric cases. It would be ideal to identify those most likely to respond. Using high-resolution microscopy, Raglianti et al. found that 12/13 slit-IgG-antibody positive steroid-resistant patients responded to second-line immunosuppression versus 2/10 slit antibody-negative [145]. Unfortunately, the high-resolution microscopy techniques required for such an evaluation are not routinely available. Perhaps such patients, as well as those with detectable circulating anti-nephrin (and other podocyte antigens) antibodies, may be considered for rituximab therapy. Specifically, for MesPGN, we also feel it is reasonable to consider rituximab for steroid-resistant cases or as first-line therapy in those wishing to avoid high-dose steroids if obvious immune-complex involvement is present, i.e., IgMN and C1qN; this recommendation is purely opinion-based and not backed by direct evidence.

## 14. Discussion

Primary MesPGN is hard to define nosologically, probably because multiple pathophysiological processes can produce that specific histology. If there is diffuse FPE and negative IFM, such cases may be classified with the primary podocytopathies, realizing that MCD, MesPGN, and FSGS are each a group of diseases and not clearly defined separate entities. Circulating factors and/or autoantibodies targeting podocyte antigens are involved in many cases. Mesangial proliferation would be secondary in such cases.

In cases with evidence for immune complex origin (IgMN or C1qN), the mesangium is the likely primary site of disease with secondary podocyte injury. IgMN and C1qN may represent primary immune complex diseases, like IgAN, although the antigenic target(s) remains to be defined. It is uncertain if the immune complexes form in situ, directed against a mesangial antigen, or form in the circulation and deposit, as in IgAN.

It remains uncertain if IgMN deserves to be considered a separate entity, as opposed to just classifying by LM. In the setting of INS, the presence of segmental sclerosis is the dominant prognostic feature, regardless of mesangial proliferation or IgM deposits. The presence of either mesangial proliferation or IgM deposits adds little to prognosis if segmental sclerosis is present, but both findings appear to impart a worse prognosis compared with MCD. Some of the studies outlined above indicate no adverse effect of IgM deposits, whereas other studies show a worse outcome; however, no study found a better outcome with IgM-positivity. The most appropriate threshold for IgM-positivity (≥1+ versus ≥2+) has not been defined.

The exact nosological position of C1qN is also unclear. Although bearing many similarities to lupus nephritis, especially when “full-house” positivity is present, there are differences. TRIs are absent and patients are negative for ANA, low complement, and anti-double-stranded DNA antibodies. Cases rarely evolve into overt SLE. It is possible, although we feel less likely, that C1qN should also be classified with the primary podocytopathies, with the positive immune deposits due to increased mesangial trafficking in the setting of severe proteinuria. Like IgMN, the presence of segmental sclerosis dominates prognosis in C1qN, but compared with MCD, C1qN has a worse prognosis.

It should be stressed that the prognostic implications of mesangial proliferation, segmental sclerosis, or immune deposits apply mainly to the time of biopsy. What ultimately determines the prognosis for the individual patient is the subsequent clinical course. Steroid responsiveness followed by no/infrequent relapses defines an excellent prognosis regardless of histology. Likewise, steroid resistance, especially if multidrug resistance, markedly increases morbidity and the risk for progressive loss of kidney function, even if MCD histologically.

The approach to treatment of MesPGN lies in the clinical presentation (see the Box 1). Non-nephrotic proteinuria and/or hematuria should be treated with general supportive care in our opinion. Immunosuppression should only be considered if nephrotic syndrome subsequently develops or if GFR declines for no other reason in a patient with significant, albeit non-nephrotic, proteinuria.

Box 1 **Key takeaways.**

Primary mesangial proliferative glomerulonephritis refers to the LM description of >3 mesangial cells per given area that is not secondary to another primary glomerulopathy (e.g., IgAN, C3G) or systemic illness (e.g., infection, SLE).

IFM of primary MesPGN may be negative or contain immune complexes (IgM +/− complement or C1q +/− immunoglobulins) and EM may or may not contain EDDs.

Primary MesPGN may present clinically as INS, non-nephrotic proteinuria +/− hematuria, or isolated hematuria.

Primary MesPGN may be classified with the primary podocytopathies (MCD, FSGS) when presenting as INS, especially in the absence of immune complexes.

In the setting of immune complexes (IgMN or C1qN), MesPGN may represent primarily mesangial disease analogous to IgAN with secondary podocyte injury.

In our opinion, the treatment of nephrotic MesPGN should mimic KDIGO guideline-based immunosuppression of the primary podocytopathies regardless of IFM (negative, IgMN, or C1qN).

Initial IS: high-dose steroids (1 mg/kg/day). Alternatives based on RCTs in the primary podocytopathies, which in our opinion are applicable to primary MesPGN: tacrolimus; possibly half-dose steroids + either MMF or alfacalcidol; rituximab.

For FR/SD primary MesPGN: extrapolating KDIGO recommendations for FR/SD MesPGN (cyclophosphamide, rituximab, CNIs, or mycophenolic acid analogs in no preferred order). Rituximab with maintenance dosing is our preferred choice unless contraindicated.

For steroid-resistant or multidrug resistant primary MesPGN: based on KDIGO guidelines and RCTs in the primary podocytopathies extrapolated to MesPGN: CNIs. In our opinion, rituximab may also be considered if concerns for CNI toxicity based on retrospective data from patients with primary podocytopathies.

For MesPGN with non-nephrotic proteinuria and/or hematuria, primary treatment should be conservative with immunosuppression possibly considered if significant non-nephrotic proteinuria (e.g., >1 g) with declining GFR for no other reason. This is purely speculative on our part.

CNI: calcineurin inhibitor; FSGS: focal segmental glomerulosclerosis; IFM: immunofluorescence microscopy; INS: idiopathic nephrotic syndrome; KDIGO: Kidney Disease Improving Global Outcomes consortium; MCD: minimal change disease; MMF: mycophenolate mofetil; RCT: randomized controlled trial; SLE: systemic lupus erythematosus.

For MesPGN patients (including IFM-negative MesPGN, IgMN, or C1qN) presenting with INS, treatment should, in our opinion, mirror that for the primary podocytopathies, as per KDIGO21 and 25 (high-dose steroids, prednisone 1 mg/kg daily). In patients wishing to avoid steroid toxicity from the outset, tacrolimus has the best supporting data extrapolated from RCTs in the primary podocytopathies. Reduced-dose steroids with either MMF or alfacalcidol have support in single RCTs for initial therapy if CNI toxicity is also a concern. There are only limited data on the safety and efficacy of rituximab as initial therapy.

In our opinion, rituximab is the preferred agent for adults with FR/SD INS regardless of LM (MCD, MesPGN, or FSGS) or IFM (IgM, C1q, or negative) using a moderate- or high-dose regimen. Pediatric guidelines prefer CNIs or MMF for FR/SD INS [160,161]. Hypogammaglobulinemia from rituximab may be of greater concern in children [162], especially young children [163], where caution is advised by pediatric KDIGO 2025 guidelines for those <7 years old or receiving multiple courses [105]. If a low-dose rituximab regimen is used, maintenance immunosuppression (CNI or MMF) should be added. A low maintenance rituximab dose should be considered (e.g., at 6 months or upon B-cell repopulation [164]), especially in those with complicated SD/FR. If there is no remission or early relapse with rituximab, obinutuzumab may be considered, especially if ARAs are detected. These recommendations are purely our opinion based on available evidence. KDIGO 2021 guidelines for adults do not favor rituximab or any other second-line agent (cyclophosphamide, CNIs, or mycophenolic acid analogs) as preferred [8].

Whereas rituximab and other B-cell depleters are reasonably well tolerated, the potential for side effects must be considered [159]. Infusion reactions are not uncommon, occurring in about one-third of patients [165], and include fever, chills, nausea, skin rash, bronchospasm, and hypotension. These can be ameliorated with premedication and a slowed infusion. Serum sickness may occur with repeated infusions in those with ARA [120]. Hypogammaglobulinemia is common (about 10% in children up to 50% or more [163]) and may occur in up to 50% of those given repeated courses [119]. Risk factors include young age, low baseline IgG, and longer maintenance immunosuppression [166]. Late-onset neutropenia occurs in < 10% of children [159], although one study found 21% [166]. Infections are not uncommon in children and adults [125] but are usually not severe. Hepatitis B may reactivate following rituximab [167] and prophylaxis in infected patients is recommended. Progressive multifocal leukoencephalopathy has been reported following rituximab but is exceedingly rare and not reported in treatment of INS; however, fulminant viral myocarditis has been reported [168].

For steroid-resistant INS, CNIs have the best data from RCTs in FSGS. High-dose dexamethasone plus MMF may be an alternative if CNI toxicity is an issue. Rituximab appears to be effective in a significant minority of cases, although at this time responsiveness cannot be adequately predicted. It remains to be determined if the presence of mesangial immune deposits (IgMN or C1qN), detection of slit diaphragm/podocyte immunoglobulins by high-resolution microscopy, or detection of circulating anti-podocyte antibodies, would increase the chance of responding to rituximab or more potent B-cell depleters.

The use of tissue, serum, and urine biomarkers for determining prognosis and as a guide to therapy remains to be explored. Li et al. evaluated the ability of various urinary proteins at time of biopsy to predict severe mesangial hypercellularity and severe segmental sclerosis in 98 children with MesPGN (40 IgAN, 37 IgMN, and 21 IFM-negative) and found the best correlation with urinary transferrin [169]. Regarding FSGS, evidence of complement activation may be such a biomarker. For example, glomerular C4d deposition may precede segmental sclerosis in both the experimental animal and in post-transplantation recurrence of FSGS [170]. Human FSGS prognosis has been associated with blood C3 and Ba levels [171] as well as the urinary C5b-9/creatinine/urinary protein ratio [172]. Trachtman et al. found that patients with primary podocytopathies (MCD or FSGS) had elevated plasma levels of C4a and soluble C5b-9 [29]. More research for MesPGN and related diseases is clearly needed.

## Data Availability

No new data were created or analyzed in this study. Data sharing is not applicable to this article.
